# Supplementary material for: The oral protease inhibitor (PF-07321332) protects Syrian hamsters against infection with SARS-CoV-2 variants of concern
Source: Nat Commun. 2022 Feb 15;13:719. doi: 10.1038/s41467-022-28354-0 (PMC8847371; doi:10.1038/s41467-022-28354-0)
Supplement: Supplementary file 1 — Supplementary Information File [file 41467_2022_28354_MOESM1_ESM.pdf]

Supplementary Information

The oral protease inhibitor (PF-07321332) protects Syrian hamsters against infection with SARS-CoV-2 variants of concern

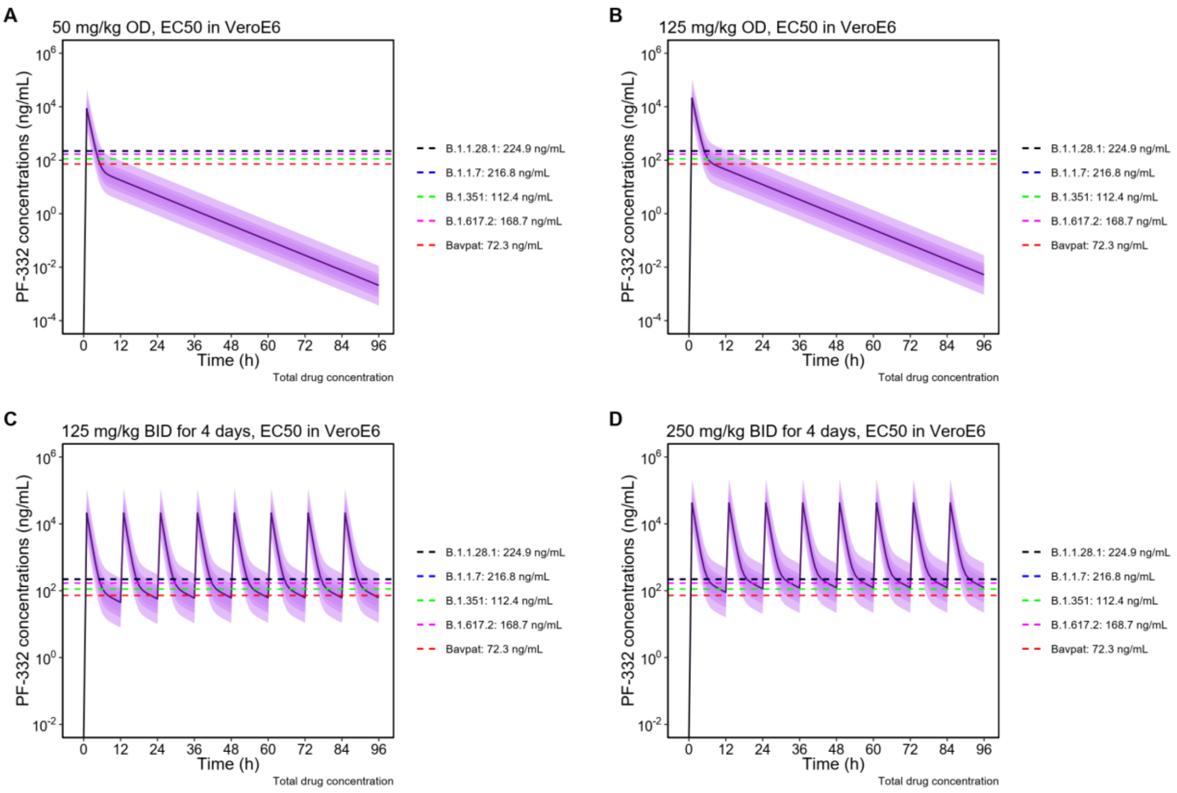

Supplementary Figure 1. The simulated concentration time profile of PF-332 with different dosing scenarios (n=1000 for each scenario) and compared with EC_{50} in Vero E6 cell line: (A) 50 mg/kg once daily, (B) 125 mg/kg once daily, (C) 125 mg/kg twice daily for 4 days, (D) 250 mg/kg twice daily for 4 days. The horizontal lines represent the EC_{50} values reported in Vero E6 cell which is corrected for 37.8% plasma protein binding in hamsters. Source data are provided as a Source Data file.

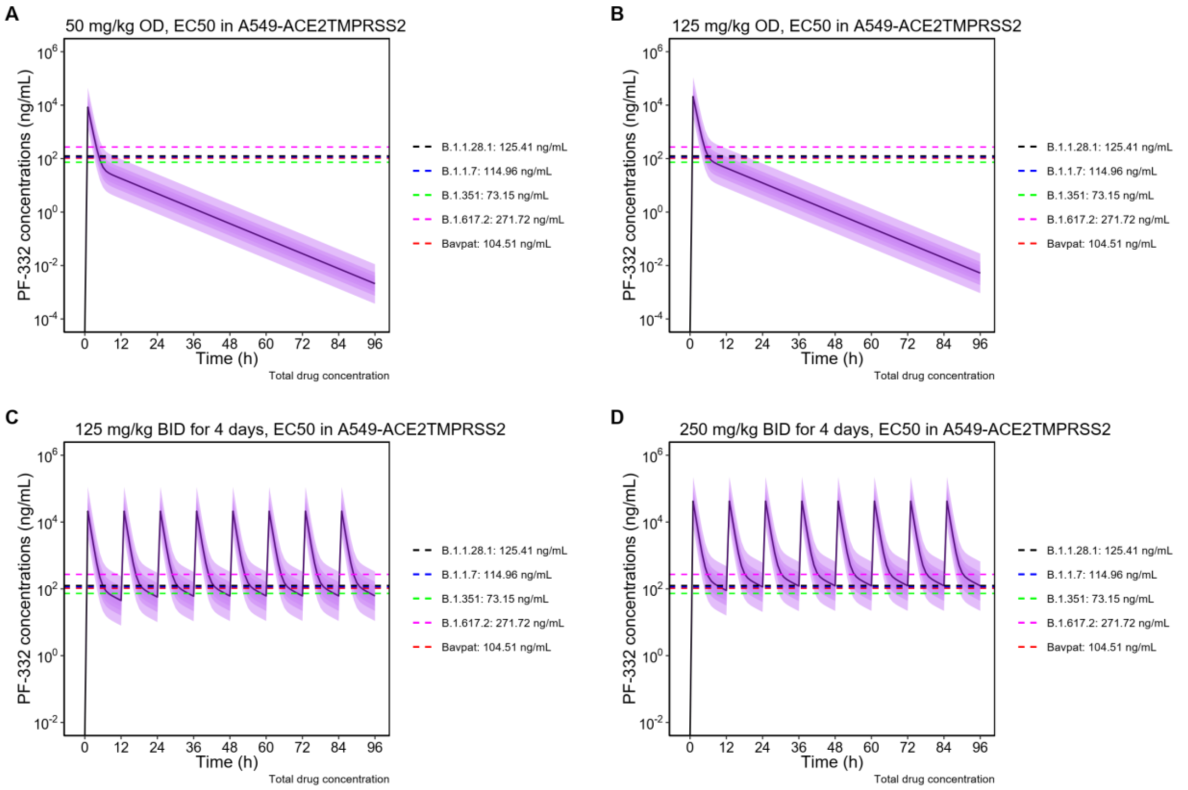

Supplementary Figure 2. The simulated concentration time profile of PF-332 with different dosing scenarios (n=1000 for each scenario) and compared with EC_{50} values obtained in A549 cell line: (A) 50 mg/kg once daily, (B) 125 mg/kg once daily, (C) 125 mg/kg twice daily for 4 days, (D) 250 mg/kg twice daily for 4 days. The horizontal lines represent the EC_{50} values reported in A549-ACE2TMPRSS2 cell which is corrected for 37.8% plasma protein binding in hamsters. Source data are provided as a Source Data file.

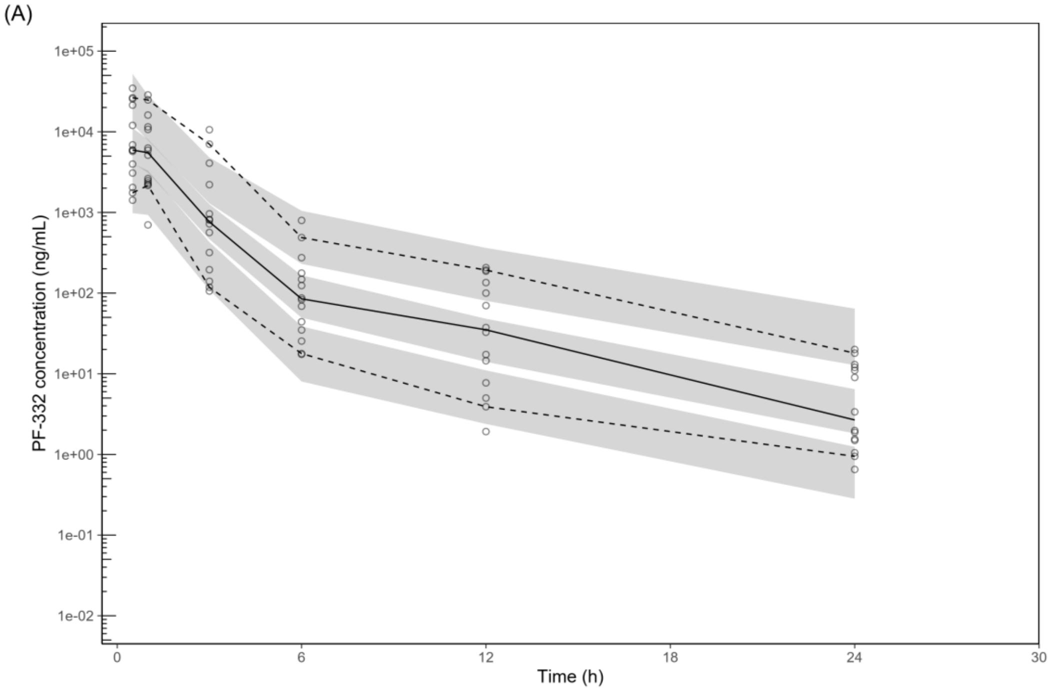

Supplementary Figure 3. Visual predictive check of the final population pharmacokinetic model of PF-332 in hamster. The open circles represent the observed PF-332 concentrations. Solid black lines represent the 50th percentiles of the observations, and dashed black lines represent the 5th and 95th percentiles of the observations. The shaded areas represent the 95% confidence intervals of each simulated percentile (n =1,000). Source data are provided as a Source Data file.

Supplementary Table 1. PF-332 plasma protein binding and microsomal metabolic stability in mouse, hamsters and human

		Mouse	Hamster	Human
Plasma protein binding	average % bound ± SD	74.0 ± 0.08	37.9 ± 6.97	45.5 ± 1.5
Microsomal stability	Modified $CL_{int, app}$ (µl/min/mg)	73.6	65.3	28.9
	Modified T-half (min)	23.5	26.6	59.9

Supplementary Table 2. Population pharmacokinetics parameters of PF-332 in hamsters

Parameter	Population estimates [a] (%RSE) [b]	95%CI[b]	IIV [a] [%CV] (%RSE) [b]	95%CI[b]
F	1 (fixed)	-	-	-
CL/F (ml/min)	9.83 (24.5)	5.88-15.5	5.40 (69.9)	0.0541 – 15.8
V/F (ml)	75.0 (89.5)	14.5-364	-	-
Q/F (ml/min)	0.292 (56.5)	0.0952-0.760	-	-
VP/F (ml)	93.5 (55.7)	31.8-250	-	-
k_a (min^{-1})	0.0215 (14.2)	0.0197-0.0303	-	-
Covariate effect compared to study 1				
Study 2-CL (% lower)	89.3 (6.15)	75.1 – 95.2	-	-
Study 2-F (% lower)	95.8 (4.65)	83.7 – 98.9	-	-
σ	0.482 (7.78)	0.303-0.562	-	-

[a] Population mean values, inter-individual variability (IIV) were estimated by NONMEM. The coefficient of variation (%CV) for IIV were calculated as $100\times\sqrt{\exp(\text{estimate})\text{ -1}}$.

[b] Relative standard error (%RSE) was calculated as $100\times(\frac{\text{SD}}{\text{Mean value}})$ from the non-parametric bootstrap results (n=1,000). The 95% confidence interval (95%CI) is presented as the 2.5 to 97.5 percentiles of bootstrap estimates.
